# Unraveling the Mixing Entropy-Activity Relationship in High Entropy Alloy Catalysts: The More, The Better?

**DOI:** 10.1021/jacs.5c15697

**Published:** 2026-01-26

**Authors:** Vladislav A. Mints, Jack K. Pedersen, John C. Olsen, Mads K. Plenge, Matthias Arenz, Jan Rossmeisl

**Affiliations:** † Department of Chemical Engineering, 4615 Imperial College London, Imperial College Rd, South Kensington, London SW7 2AZ, U.K.; ‡ Center for High Entropy Alloy Catalysis (CHEAC), Department of Chemistry, 4321 University of Copenhagen, Universitetsparken 5, 2100 København Ø, Denmark; § Department for Chemistry, Biochemistry and Pharmaceutical Sciences, 111306 University of Bern, Freiestrasse 3, 3012 Bern, Switzerland

## Abstract

The variety of publications reporting high-entropy alloy (HEA) catalysts with exceptional activities creates a survivor bias, implying that the mixing entropy directly increases the activity. However, many screening studies show a different picture. In a multielement composition-activity space, often a low to medium entropic 2- or 3-element composition emerges as the most active catalyst. In this work, we investigate the relationship between the complexity of an alloy, which can be expressed in mixing entropy, and its maximum possible activity using theory and statistical modeling. Based on our analysis, we propose a hypothesis for the surface complexity-activity relationship of HEA catalysts. Namely, the intrinsic activity of an alloyed surface is defined by two opposing forces: positive ligand interactions that enhance the activity and the statistical dilution of active sites. As a result, the relationship between the surface complexity-activity shows qualitatively a volcano-like behavior. At first, adding elements increases the activity due to favorable ligand interactions. Yet, at some point, the catalytic benefit from increasing the complexity of the surface gets outweighed by the dilution of the catalytic sites. Correspondingly, this hypothesis states that there is an optimal ratio between the surface complexity and catalytic activity.

## Graphical abstract

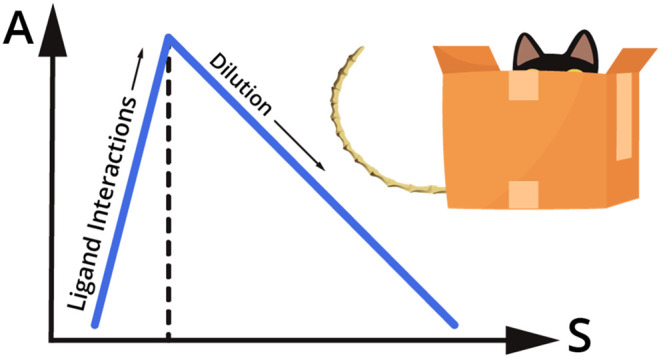

## Introduction

The field of high-entropy alloy (HEA) catalysis is receiving significant attention, as evidenced by the exponential number of appearing publications.^1^ HEAs were proposed by Yeh et al.^2^ and Cantor et al.^3^ as a class of alloys that have a mixing entropy larger than 1.5 R, which is observed for solid solutions composed of at least 5 different elements. In this definition, the surface layer responsible for the catalytic activity is generally assumed to match the bulk composition. Consequently, these highly disordered surface structures, composed of diverse atomic arrangements, provide a powerful platform for catalyst discovery by enabling precise tuning of adsorption energies for catalytic intermediates.^4^ This sparked the hypothesis that HEA can yield novel catalysts that can replace conventional catalysts. In particular, the early report of Löffler et al.^5^ demonstrated that the widely studied Cantor alloy,^3^ Co_0.2_Cr_0.2_Fe_0.2_Mn_0.2_Ni_0.2_, has a high affinity to the oxygen reduction reaction (ORR). Many publications have showcased HEA catalysts that rival conventional catalysts.^6–9^ However, the large body of auspicious results contributed to a survivor bias, reinforcing a self-fulfilling prophecy that HEAs are inherently exceptional catalysts.

The variety of different elements in a HEA catalyst provides an unexplored material library containing 10^78^ possible alloy compositions.^10^ Hence, exploring all possible compositions is not only an impossible task but also unnecessary. Instead, research should focus on identifying and studying the most promising compositions. Typically, these are catalysts that exhibit surface sites with exceptional activity relevant to industrial applications. As a result, exploration studies have become essential to chart large composition-activity landscapes, allowing researchers to rationally select the most interesting compositions for further investigation.^11–14^

Efficient exploration of large composition-activity landscapes is facilitated by high-throughput methods, which have seen significant developments over recent decades.^15^ Early work focused on developing synthesis methods for material libraries, which consist of systematically varied compositions prepared on a single substrate. One of the initial approaches involved cosputtering from a multielement target, resulting in continuous composition gradients across the substrate.^16^ Alternatively, by introducing masks during the sputtering, pixels with discrete compositions could be constructed.^17–19^ Additionally, inkjet printing has been investigated, where solutions of precursor salts are deposited and subsequently calcined to form the materials of interest.^20^

With the appearance of material libraries, complementary high-throughput activity measurements were developed. One of the first methods relied on fluorescent pH indicators to visualize catalytically active regions on the material library.^20^ Alternative methods, included the use of individual current collectors for each sample region, enabling parallel electrochemical testing in a single cell.^21^ However, performing such electrochemical testing in a shared electrolyte introduces the risk of crosstalk during extended measurements. To mitigate this, multichambered electrochemical cells were developed, isolating each sample in a separate compartment.^22^ Alternatively, scanning electrochemical probes have been employed to analyze each region at a time.^23,24^

These developments created the scanning droplet cell, which currently is the state-of-the-art high-throughput system for HEA catalyst investigations.^25,26^ At present, it has been successfully employed to study several composition-activity spaces for different reactions. For example, this method has been used to investigate Co–Cr–Fe–Mn-Ni^27^ and Ti–Ni–Cu–Zr–Pd-Hf^28^ spaces for the hydrogen evolution reaction (HER). In Co–Cr–Fe–Mn-Ni, the most active composition was proposed to be Co_56_Cr_8_Fe_19_Mo_7_Ni_10_, whereas in Ti–Ni–Cu–Zr–Pd-Hf, the most active composition was Ti_11_Ni_13_Cu_18_Zr_17_Pd_19_Hf_22_. Similarly, Ti–Ni–Cu–Zr–Pd-Hf,^28^ Ru–Rh–Pd–Ir-Pt,^29^ and Ag–Ir–Pd–Pt-Ru^25^ were studied for the oxygen reduction reaction (ORR). In these studies, the most active catalyst compositions were proposed to be Ti_14_Ni_17_Cu_16_Zr_21_Pd_17_Hf_15_, Ru_25_Rh_15_Pd_31_Ir_15_Pt_14_, and Ag_5_Ir_5_Pd_17_Pt_68_Ru_5_, respectively. Lastly, in the Co–Cr–Fe–Mn-Ni,^30^ space (Cr_25_Mn_16_Fe_17_Co_26_Ni_16_)­O_*x*_ was the most active composition for the oxygen evolution reaction (OER). It should be noted that among the identified optima, indeed, some do not fulfill the criteria to be classified as a HEA. Others are located on the edges of the explored spaces, suggesting that they can further be improved by going beyond the observed edge.

Besides grid studies, Bayesian optimization emerged as the state-of-the-art optimization algorithm. In the work of Pedersen et al.,^12^ researchers benchmarked Bayesian optimization on density functional theory (DFT) models describing the ORR activity of Ag–Ir–Pd–Pt-Ru and Ir–Pd–Pt–Rh-Ru spaces. Both models exhibited optima at Ag_20_Pd_80_ and Ir_10_Pd_55_Rh_5_Ru_30_, respectively, which do not classify as HEA. Due to the relatively smooth mathematical landscape of these models, it took on average less than 50 experiments to identify the optimum in either space. This low number of experiments makes Bayesian optimization suitable to optimize HEA catalysts via a medium-throughput approach, i.e., testing around 100 to 200 different catalysts. In the work of Xu et al.,^31^ researchers expanded the idea by using the Bayesian optimization algorithm to optimize a HEA composition for multiple objectives. Namely, they searched for the Pareto-Front of the Ag–Au–Cu–Ir–Os-Pd–Pt–Re-Rh-Ru for the mixing entropy, ORR activity, and raw material price. Interestingly, the Pareto Front between the mixing entropy and activity revealed an inverse relationship. As the mixing entropy increases, the optimal composition becomes less active.

Combining all of this empirical evidence leads to the inevitable question: Can HEA outperform optimized conventional low-entropy alloy catalysts? To answer this question, we discuss the theory behind alloy catalysis. Then, we will analyze the catalyst composition from a statistical view, which will give insight into some design principles. Combined, this discussion produces a hypothesis on the relationship between alloy complexity, which is expressed in mixing entropy, and maximum catalytic activity.

## Theory Behind Alloy Catalysts

The theory describing the intrinsic catalytic activity of a HEA^4^ is based on the well-established descriptor-based approach. The intrinsic catalytic activity is defined as the activity at standard electrochemical conditions free from extrinsic factors including mass transport, poisoning effects, and side reactions. This theory also employs the previously discussed definition of HEAs, where the surface composition is assumed to match the bulk. This approach correlates the adsorption energy of catalytic intermediates to the measured catalytic activity.^32–35^ As this correlation is expected to follow the Sabatier principle, a volcano-shaped relationship is expected, which is typically modeled with eq 1.^33^ In this eq, Δ*E* is the adsorption energy of the catalytic intermediate, Δ*E*_opt_ is the optimal adsorption energy, *k*_B_ and *T* are the Boltzmann constant and temperature, respectively.

1 $$a\left(\Delta E\right)=e^{-\left|\Delta E-\Delta E_{\mathrm{opt}}\right|/k_{B}T}$$

While high-entropy alloys provide a diverse set of binding environments, the fundamental constraints of the Sabatier principle still apply. For reactions involving a single adsorbing element—such as oxygen in the oxygen reduction reaction (ORR), hydrogen in the hydrogen evolution reaction (HER), or nitrogen in the nitrogen reduction reaction (NRR)—the catalytic activity is ultimately governed by the adsorption and desorption energy of an intermediate of that element. This leads to a volcano-type relationship, which in this work is assumed to result from a simplified reaction mechanism, where the rate is limited by adsorption and desorption steps. While real catalytic systems may involve more complex pathways, the volcano used here reflects an intrinsic activity model based on this minimal yet necessary mechanistic constraint. Although surface species may diffuse between sites with different binding affinities, this does not circumvent the inherent thermodynamic limitations: the energy required for diffusion from a strongly binding site to a weakly binding site is equal to the energy difference in direct adsorption between these sites. As such, the activity trends remain constrained by the Sabatier principle. In the case of a pure metal surface, all catalytic sites are identical, so the total activity can be represented with the activity of a single site. Therefore, pure metal surfaces can produce only discrete adsorption energies and activities. However, the formation of an alloy introduces novel active sites on the surface with perturbed adsorption energies. This creates a variety of adsorption energies at the surface, resulting in an adsorption energy distribution (*f*(Δ*E*)). Using eq 2, this adsorption energy can be transformed into an activity distribution *A*(Δ*E*). Hence, the total activity of a catalyst, given by eq 3, is a linear combination of all of the present activities or, equivalently, the integral of the activity distribution. In this Equation, *i* is the index of a unique site, and *P* is the probability of finding site *i*. This process is schematically depicted in Figure 1.

2 A(ΔE)=a(ΔE)·f(ΔE)

3 $$A=\sum_{i}a\left(\Delta E_{i}\right)\cdot P\left(i\right)=\int_{-\infty }^{\infty }a\left(\Delta E\right)\cdot f\left(\Delta E\right)d\Delta E$$

We have earlier shown that the adsorption energy distribution for a single element in an alloy can be approximated by a normal distribution.^36,37^ Thus, the adsorption energy distribution of an alloy is represented with the sum of all individual normal distributions weighted by the element concentration, as described by eq 4. In this eq, *n* represents the number of elements in the alloy, *P*(*m*_*i*_) denotes the probability of finding metal at number *i* on the surface, *N* is the normal distribution. The parameters $\overset{®}{\Delta E_{m}\;}$ and σ_*m*_ correspond to the mean and the standard deviation of the adsorption distribution for element *m*_*i*_.

4 $$f\left(\Delta E\right)=\sum_{i=1}^{n}P\left(m_{i}\right)N\left(\overset{®}{\Delta E_{m_{i}\;}\;},\sigma_{m_{i}}\right)$$

**Figure 1:**
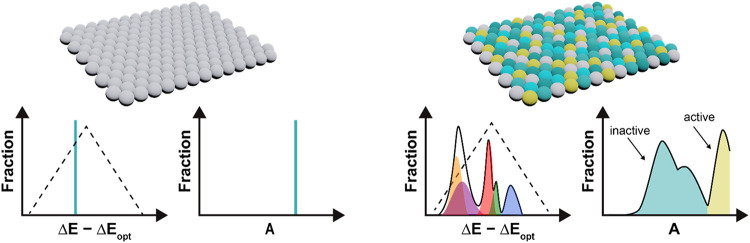
A schematic representation of how the adsorption energy distribution and activity distribution are affected by the alloying process.

According to this model, three primary effects can be used to tune the adsorption energy distribution of an alloy, as schematically depicted in Figure 2. The first and most important effect is peak widening, which mathematically corresponds to an increase in σ_*m*_. This effect arises from the formation of a variety of distinct active sites during the alloying process. These sites differ due to variations in their local atomic environments, where unique atomic configurations and ligand perturbations give rise to a range of adsorption energies. As a result, the adsorption energy distribution broadens. The width of this distribution is primarily determined by the strength of the ligand perturbations; stronger perturbations lead to a wider spread in adsorption energies. Additionally, the number of different elements in the alloy increases the diversity of possible local environments, which further contributes to the broadening. The number of atoms involved in the active site and its immediate surroundings also plays a role, as larger or more complex sites allow for more configurational variation. In other words, the width of the adsorption energy distribution qualitatively scales with the configurational entropy of the system: higher entropy (e.g., near-equimolar compositions) corresponds to a greater variety of adsorption sites and thus a broader distribution. Generally, this process creates active sites with adsorption energies closer to the optimal adsorption energy, leading to an enhanced activity. However, if peak widening is excessively strong, it may cause the adsorption energies to overshoot the optimum, ultimately reducing the activity. The second effect is the ensemble shift, which corresponds to changes in the mean adsorption energy $\overset{®}{\Delta E_{m}\;}$ . In addition to broadening the distribution, ligand interactions can alter the average adsorption energy of the element. Shifts that move the mean adsorption energy closer to the optimum adsorption energy enhance the catalytic activity, whereas shifts in the other direction reduce it. The third effect is the dilution of the element, which is expressed through a decrease in *P*(*m*). As the number of elements in the alloy increases, the fraction of each element on the surface decreases. Hence, it is an entropy-driven term that reduces the fraction of the most active sites, contributing to a decrease in activity. What makes the field of alloy catalysis challenging and exciting is that the effects are interdependent. Adjustments to one factor influence the others, necessitating careful balance to achieve optimal catalytic performance. Designing an alloy catalyst therefore requires identifying the ideal composition that optimally balances these effects.

**Figure 2:**
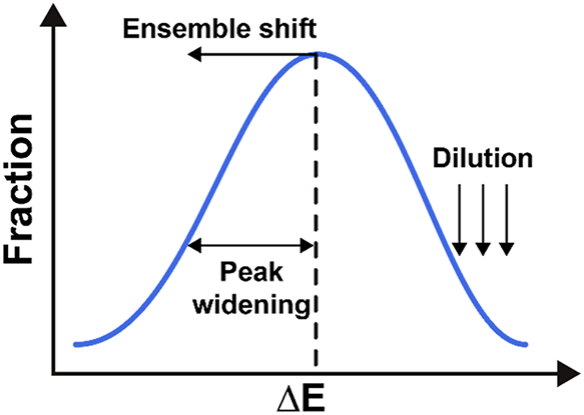
A schematic depiction of the three primary effects that influence the adsorption energy distribution.

Due to the vast composition space of HEAs it is impossible to investigate all possible compositions and their adsorption energy distributions. However, mathematical explorations allow us to analyze trends and limits between the variables. Here, we illustrate the relationship between the catalytic activity and $\overset{®}{\Delta E_{m}\;}$ for equimolar compositions. In this analysis, we assume that only one element is catalytically active, while the other elements contribute exclusively to ligand interactions. Additionally, we assume that σ_*m*_ scales linearly by 0.5 with the number of additional elements. The resulting adsorption energy distributions for this model are described in eq 5.

5 $$f\left(\Delta E\right)=\frac{1}{n}N\left(\overset{®}{\Delta E_{m}\;},0.5\left(n-1\right)k_{B}T\right)$$

The trends derived from this model are presented in Figure 3. This Figure illustrates that as the number of elements goes up, the maximum achievable activity of the ensemble goes down because the active element is diluted. The model suggests that there are threshold values $\overset{®}{\Delta E_{m}\;}$ at which the activity of an alloy with n elements will equal the maximum activity of one with *n+1* elements. These thresholds are shown with dashed lines in Figure 3. At Δ*E* smaller than this threshold, it cannot be beneficial to alloy, but at Δ*E* larger than these thresholds, alloying could be beneficial. As σ_*m*_ is artificially defined, we will refrain from overinterpreting these threshold values. Yet, their existence showcases that scenarios exist where increasing the number of elements in an alloy cannot result in an increase in activity. Figure 3 shows that if a metal with a $\overset{®}{\Delta E_{m}\;}$ close to the Δ*E*_opt_ exists, then the most active composition will correspond to a low-entropic material. Conversely, a high-entropy composition will exhibit maximum activity only if all constituent elements individually exhibit poor catalytic performance.

**Figure 3:**
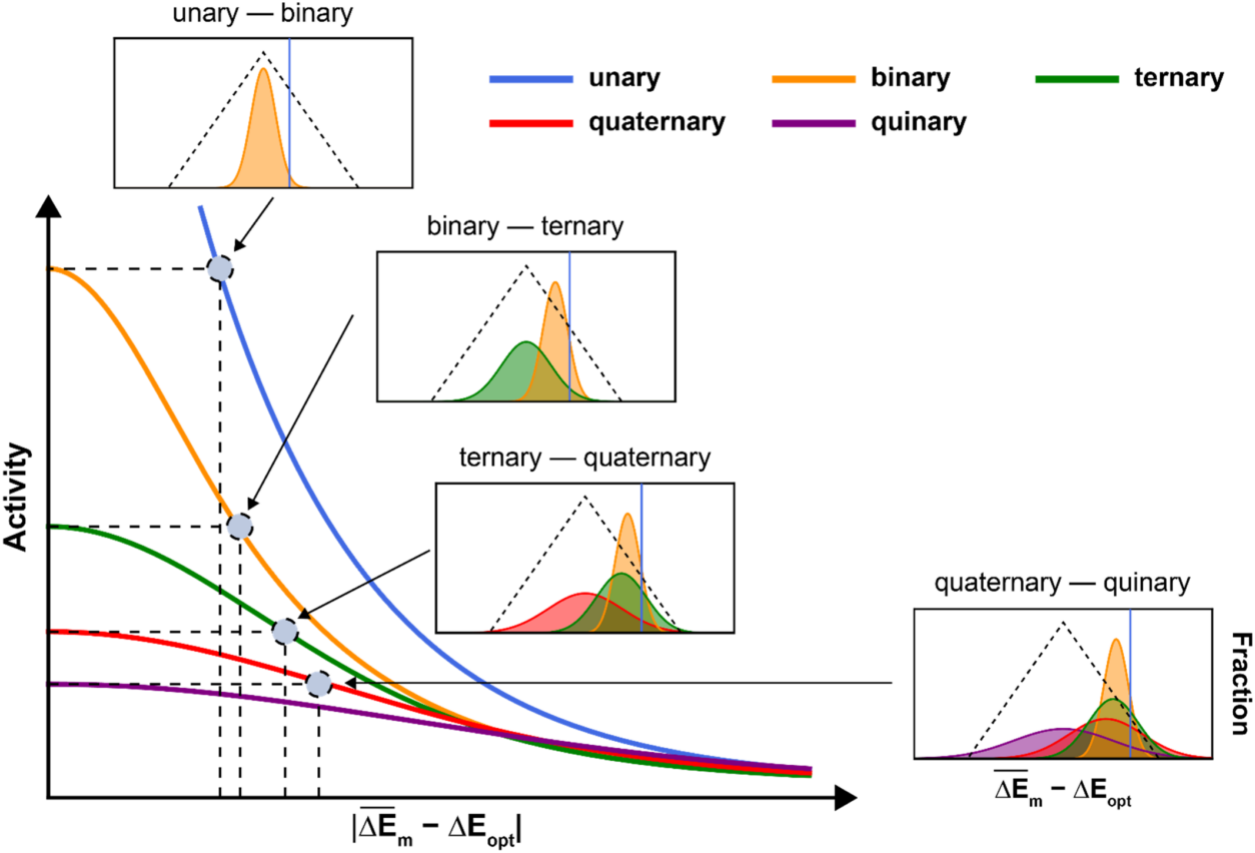
Relationship between $\overset{®}{\Delta E_{m}\;}-\Delta E_{m}$ and the activity for equimolar catalysts. The *x*-axis represents the difference between the optimal adsorption energy and the mean of the adsorption energy distribution for each catalyst. The dashed lines indicate the critical points at which the activity of an *n*-element catalyst equals the maximum activity of an (*n*+1)-element catalyst. The inserts show the adsorption energy distributions for equimolar catalysts at these critical points that would lead to the same total activity.

Additionally, this calculation highlights the properties of the peak widening effect, which is expected to scale with the mixing entropy. Specifically, the wider the adsorption energy, the more resilient the catalyst activity becomes to the ensemble shift. In the limit, the normal distribution can be approximated by a constant, and the total activity becomes linearly dependent on the element concentrations (*P*(*m*)). As a result, the partial derivative of the activity with respect to the element concentration (*δA*/*δP*(*m*)) is approximated by a constant whose magnitude depends mostly on the intrinsic activity of the differentiated element. Even though this is a mathematical extreme, it suggests that high-entropy composition-activity landscapes are more likely to exhibit a linear character compared to low-entropy composition spaces. Consequently, composition-activity spaces that demonstrate step changes would be indicative of the presence of surface ordering. In part, this may contribute to why HEA composition-activity spaces experimentally exhibit smooth mathematical landscapes.^12,38,39^

## Statistical View on Alloy Catalysis

Alternatively, the alloying process can be analyzed from a purely statistical perspective. Alloying introduces ligand elements whose primary role is to enhance the activity of the primary element through the ligand interactions. “Ligand interactions” is here used as an umbrella term for the peak widening and ensemble shift effects. Physically, this corresponds to the formation of the complex *n-*element catalytic sites. However, these additional elements also occupy a fraction of the catalytic surface, effectively diluting the presence of the primary metal. Since these ligand elements are generally less catalytically active, the fraction of the surface they occupy can be approximated as inactive or lost (Figure 1). This introduces a constraint on the design principles of an alloy catalyst. Namely, designing an alloy catalyst is beneficial only if the formed complex *n*-element sites can compensate for the surface area investment.

In a disordered alloy, to which HEAs belong, the bulk and surface structures are identical and random. Hence, the probability of finding a specific active site is governed solely by statistics. Thus, by constructing a statistical model, it is possible to estimate the catalytic site distributions for different alloy compositions. This distribution, in turn, enables estimation of the minimum activity that complex sites must achieve for the alloying process to be viable. The statistical model we designed to estimate these minimum activity thresholds is outlined in Table 1 and Figure 4.

**Figure 4:**
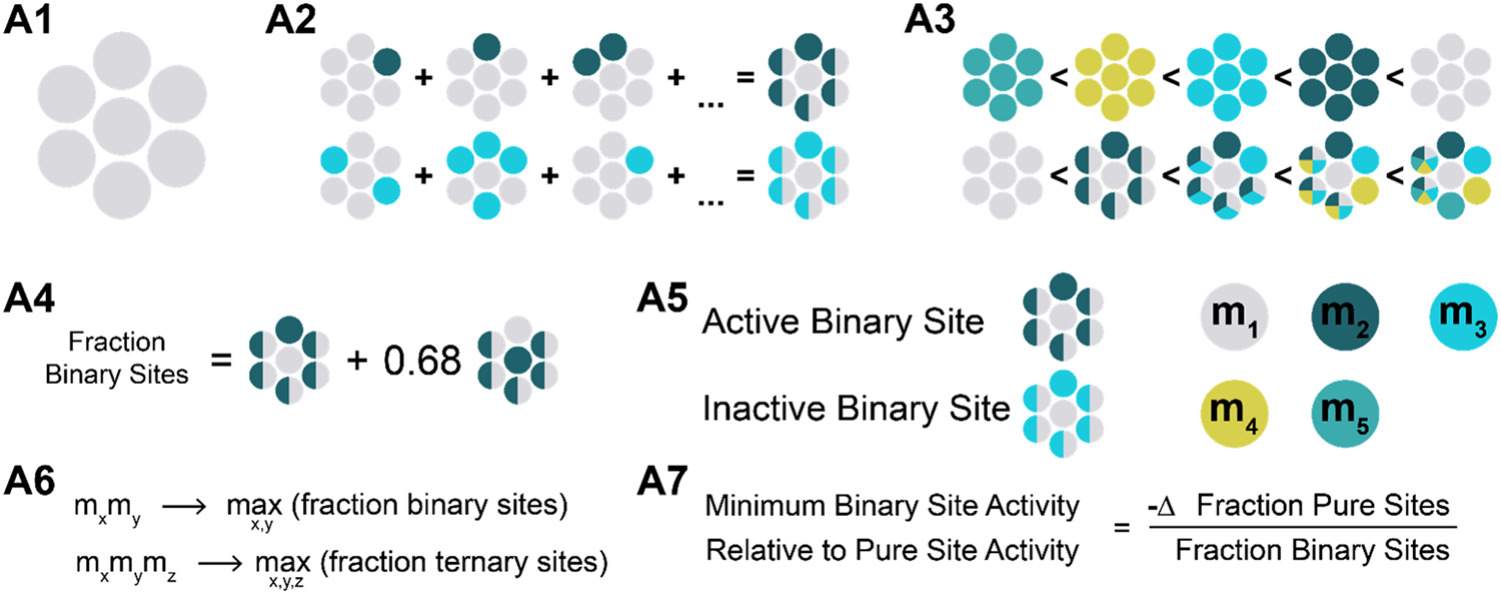
A visual explanation of the assumptions in the statistical model.

**Table 1:** List of Assumptions of the Statistical Model

**A1**	a catalytic site is defined by a center atom and six neighbors.
**A2**	the activity of a catalytic site is defined by the center atoms and the distinct atoms. Thus, all permutations and combinations of the same atoms have the same activity.
**A3**	the activity of the sites increases with increasing the number of distinct atoms in the neighborhood.
**A4**	catalytic sites with m_1_ in the center are considered fully active. Catalytic sites containing m_2_ in the center are considered 32% less active than the catalytic sites containing m_1_ in the center. The rest of the sites are assumed to be inactive.
**A5**	in a complex site, only the most active combination, which appears first during the alloying process, is considered active. The rest of the combinations are considered inactive.
**A6**	the composition should maximize the fraction of the most active catalytic sites.
**A7**	the most complex catalytic sites need to compensate for the loss in surface area of the less complex sites with their increased activity.

The first assumption in the model (A1) is the definition of the catalytic site that will be modeled. In this model, the catalytic site is defined as a 7-atom arrangement consisting of one central element surrounded by 6-elements. This catalytic site configuration resembles an on-top site on a (111) surface, which is typically used in DFT calculations.^4^ Compared to bridge sites and hollow sites, this type of arrangement has a core composed of only a single atom and 6-elements occupying equivalent positions. This symmetry simplifies the mathematical treatment of this active site, making it a convenient choice for this model. For disordered alloy surfaces, incorporating additional complexity into this model—such as including subsurface neighbor atoms, effectively increases the number of neighboring atoms without altering the qualitative conclusions drawn from this model.

The second assumption (A2) focuses on grouping catalytic sites based on their associated activity. Specifically, the activity of a catalytic site is defined by the center atom and the distinct atoms in the 6-element surrounding. For example, a binary alloy can be described by four types of catalytic sites, whereas a ternary alloy is described by 12 sites.

The third assumption (A3) is used to rank the catalytic sites based on their activity without assigning specific numerical values. It states that the more complex a catalytic site, the more active it will become. The necessity of this assumption is to shape a scenario in which designing a HEA catalyst is beneficial. After all, if the more complex site is less active, there is no incentive to invest in its formation, from an activity point of view. This assumption also determines the sequence of alloying. Namely, the first metal (*m*_1_) in the system is the most active metal. The second added element m_2_ forms the most active binary site (*m*_1_ *m*_2_). Similarly, the third added element (*m*_3_) forms the most active ternary site (*m*_1_ *m*_2_ *m*_3_), and so on.

The fourth assumption (A4) simplifies the analysis further by reducing the number of catalytic sites that must be considered. According to this assumption, only active sites with the primary element (*m*_1_) in the core are considered active, while active sites with the second element (*m*_2_) are considered 32% less active than their m_1_-equivalent. The rest of the catalytic sites are considered inactive. The difference in 32% is estimated from the descriptor-based approach using eq 1 and corresponds to a difference in adsorption energy of 0.01 eV under standard conditions. Subsequently, *m*_1_ and *m*_2_ sites with identical complexity can be summed using a weighted sum.

The fifth assumption (A5) is introduced to prevent artifacts where substituting an element is always beneficial. It separates the complex sites into active and inactive components. The active part is composed of the complex site that first appears during the alloying process. Thus, these active binary, ternary, quaternary, and quinary sites are composed of *m_1_m*_2_, *m*_1_ *m*_2_ *m*_3_, *m*_1_ *m*_2_ *m*_3_ *m*_4_, and *m*_1_ *m*_2_ *m*_3_ *m*_4_ *m*_5_, respectively. Other combinations, such as binary site *m*_1_ *m*_3_, are considered inactive.

The sixth assumption (A6) defines which compositions will be investigated. In this study, the decision was to investigate the best-case scenario for the most complex site. Therefore, the compositions are chosen to maximize the fraction that the most complex sites occupy, with the fraction calculated using assumption A4. Combining assumptions A1-A6, we can estimate the catalytic site distributions in Figure 5. This Figure shows that the maximum fraction that the most complex sites occupy decreases as the number of elements increases. For instance, 2-element sites can at most occupy 84% of the surface, whereas 5-element sites can only occupy 7% of the surface.

**Figure 5:**
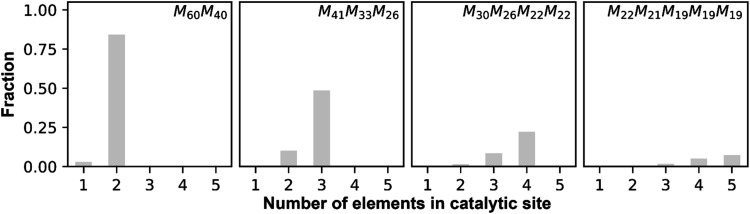
Active catalytic site distributions as calculated using the statistical model.

The final assumption (A7) is used to estimate the minimum activity that complex sites need to possess for an alloy to be directly competitive with its less complex components. Thereby, it is assumed that only the most complex site must compensate for the surface loss. Thus, this minimum activity can be calculated by taking the difference in surface area dedicated to a specific type of active site and dividing it by the surface area that the most complex site occupies. Performing these calculations for all catalytic site pairs produces Figure 6. It shows that for a HEA, 5-element sites need to exhibit at least 13.6 times the activity of the pure metal sites to outperform them directly. Additionally, the five-element sites must outperform 2-element sites by a factor of 11.5, 3-element sites by a factor of 6.4, and 4-element sites by a factor of 2.3. If all of these conditions are met, the HEA emerges as the most active alloy. Yet, if one of the conditions is not fulfilled, maximizing the fraction of 5-element sites stops being favorable. Depending on how much the 5-element sites underperform, they may either act as minor tuning sites that slightly boost the activity or lead to a decrease in activity by diluting the active catalytic sites.

**Figure 6:**
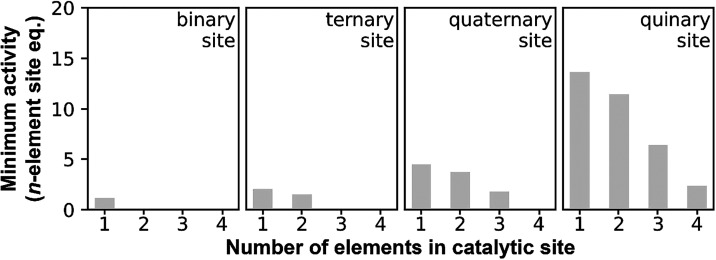
Minimum activities that catalytic sites need to possess to directly compensate for the surface loss of their less complex components.

This statistical model purely investigates the relationship between the number of catalytic sites and the number of elements. Thus, it is independent of any theoretical framework but can be combined with one to analyze which reactions may benefit from HEA catalyst design. Here, we combine it with the descriptor-based approach to analyze the widely studied ORR. According to the descriptor-based approach, it is known that the OH adsorption energy on Pt is 0.1 eV stronger than the ideal adsorption energy.^40,41^ Using eq 1, which describes a thermodynamic volcano, the activity of an ideal ORR catalyst is equal to 48 Pt equivalents (Pt eq). Assuming that all of the most complex sites are ideal, it is possible to calculate the maximum activity increase due to them. Here, a decreasing trend is observed: a 2-element site can increase the activity of Pt by a factor of 40, whereas a 5-element site can only increase the activity by a factor of 3.5 (Figure 7). Furthermore, using the available data, it is possible to estimate which binary and ternary alloys will never be outperformed by a HEA. These correspond to alloy activities of 6.4 and 5.6, respectively. The work of Stamenkovic et al.^42^ demonstrates that a Pt skin on Pt_3_Ni­(111) outperforms Pt(111) by a factor of 10, an activity that, according to this analysis, HEAs can never directly outperform. Furthermore, the upper limit of 48 Pt eq is calculated using the thermodynamic volcano, which may be substituted with the kinetic volcano.^43^ The kinetic volcano has a flatter peak compared to that of the thermodynamic volcano, resulting in a lower theoretical maximum activity. Consequently, as the room for improvement shrinks, according to the model, it becomes impossible for HEA catalysts to outperform Pt_3_Ni­(111) and improbable to outperform pure Pt.

**Figure 7:**
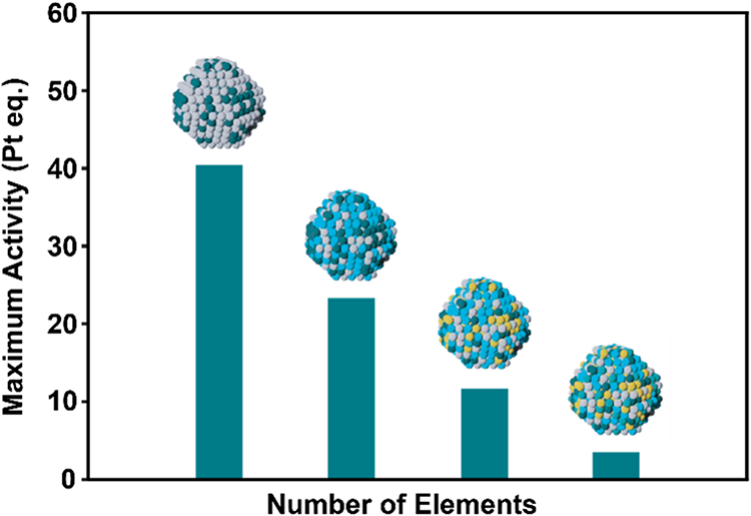
Maximum ORR activity originating from only the most complex sites in different *n*-element alloys.

As the statistical model stands separately from any catalytic reactions, the same analysis can be applied to other reactions. The prerequisite would be that the theoretical maximum activity of the reaction is known. Despite assumption A1 resembling an on-top (111) site, this statistical model could serve as an approximation for reactions that require multiple elements. The key difference to the ORR analysis would be that the most active catalyst is initially a 2-element system rather than a pure metal. If necessary, then the statistical model can be further tailored to be more specific for a particular reaction. However, given that the statistical influence on the surface fractions remains similar, we expect the results to be comparable.

Similarly, the conclusions from this statistical analysis still hold if transition states are included in the theoretical calculations. A transition state energy can be viewed as the binding energy of a distorted molecule. Unlike the on-top adsorption configuration assumed in the statistical model, transition states may bind to multiple surface atoms. This effectively increases the size of the catalytic site and the number of possible permutations. Because the dilution effect is driven by the number of permutations, it is expected to scale proportionally with this increase.

## Alloy Complexity – Activity Relationship

Based on these considerations, we can provide a hypothesis for the surface complexity-activity relationship. The total activity of a disordered catalytic surface is primarily defined by two opposing forces (Figure 8a). On one hand, favorable ligand interactions enhance the catalytic activity.^44^ On the other hand, dilution reduces the fraction of the catalytically active surface. In systems where the bulk composition equals the surface composition, surface complexity can be qualitatively expressed by the alloy’s mixing entropy. Thus, in low mixing entropy alloy catalysts, the total activity is primarily defined by ligand interactions that may favor alloying. However, as the complexity of the alloy and the mixing entropy increase, the effect of statistical dilution becomes more pronounced. At a certain point, a tipping point is reached, where beneficial ligand interactions can no longer compensate for the dilution effects. This tipping point, which balances these two opposing forces, forms the optimal mixing entropy, as here the most active catalytic surface is found.

**Figure 8:**
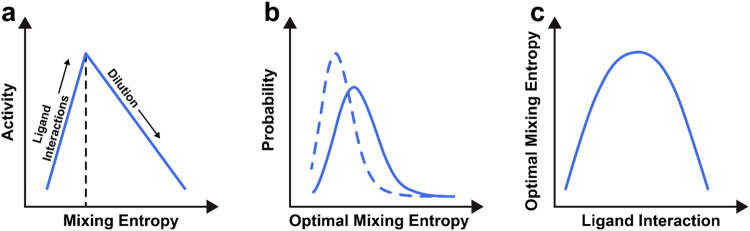
A qualitative view on the properties of the optimal mixing entropy. (a) Relationship between the activity and mixing entropy. (b) Qualitative relationship describing the probable optimum mixing entropy. The dashed line shows a situation where one of the pure metal components has an adsorption energy close to the optimum. The solid line shows the situation where all pure metal components are by themselves terrible catalysts. (c) Qualitative relationship between the strength of a ligand interaction present in the composition space and the optimal mixing entropy.

This optimal mixing entropy depends on the composition space that is being investigated, and it can be only experimentally identified. However, it does exhibit a few general properties schematically depicted in Figure 8b,c. First, the optimal mixing entropy appears to be qualitatively dependent on the elements spanning the composition space. If the chosen elements are all intrinsically terrible catalysts, then there is great room for improvement, which increases the maximum influence that the ligand interactions can introduce before they succumb to the statistical dilution. Hence, we can qualitatively represent the probability density function of the optimal mixing entropy as a function of the “room of improvement” as shown in Figure 8b. Second, the optimal mixing entropy also depends on the magnitude of the ligand interactions, as depicted in Figure 8c. If these interactions are weak, they will have a limited impact on enhancing the activity, and the dilution will dominate quickly. Conversely, if the ligand interactions are very strong, the formation of ideal catalytic sites will occur rapidly shrinking the “room for improvement”. Thus, only if all ligand interactions are moderate, the optimal mixing entropy may be high entropic.

In summary, these implications suggest that a HEA composition can emerge as the most active composition in its composition space only if both criteria are met. The HEA composition space must be composed of intrinsically poor catalysts, and the elements must exhibit moderate synergistic ligand interactions. The likelihood for such a system to exist appears intuitively low. In addition, as demonstrated previously, the dilution effect scales with the mixing entropy of the alloy. Consequently, the maximum achievable activity of an alloy system is inversely related to its surface mixing entropy. Thus, the theoretical maximum possible intrinsic activity achievable by a HEA is lower than the maximum possible activity of a low-entropic alloy.

This hypothesis on the mixing entropy-activity relationship allows us to revisit optimization strategies for alloy catalysts. Classically, the bottom-up approach is used, where catalyst complexity is gradually increased by sequentially adding elements. Within the context of the mixing entropy-activity relationship, this approach evaluates the activity-composition landscapes where activity is predominantly shaped by the nonlinear and hard-to-predict ligand interactions. Consequently, this approach necessitates testing all possible elemental combinations to ensure that no significant ligand interactions are overlooked. Alternatively, we proposed in the work of Mints et al.^38^ to invert this bottom-up approach and instead start with a complex multielement system. In this method, elements with a minimal influence on the catalytic activity are progressively removed, simplifying the system until the optimal catalyst is identified. This approach starts in the high-entropy regime, where statistical dilution is the primary force defining the activity. Unlike ligand interactions, the statistical dilution is mathematically a relatively smooth function that produces a smooth composition-activity landscape. This makes the modeling of high-entropy composition-activity spaces computationally cheap, requiring little experimental data.^12,31,38,39^ In addition, as the high-entropy alloy composition space falls within the statistical dilution regime, the only way to enhance the catalytic activity is to reduce the mixing entropy through the elimination of elements. Therefore, by following the gradient of activity within the high-entropy composition space, it becomes possible to navigate systematically toward the most active low-entropy regime. This eliminates the need for testing all possible combinations, as required by the classical approach, and provides a more streamlined pathway for optimizing alloy catalysts.

Furthermore, this hypothesis allows us to question the widely praised “cocktail effect”,^45^ which states that the total activity of an alloy catalyst may be higher than the sum of its individual parts. Currently, this effect is widely used to explain the high activity observed for HEA catalysts. However, we argue that in the high-entropy regime, the statistical dilution effect dominates the ligand interactions. As a result, the enhanced activity of a HEA catalyst is unlikely to stem from the formation of highly active, complex catalytic sites. This is because such sites, while potentially highly active, are statistically rare on the surface and therefore contribute only marginally to the total activity. Thus, we expect that in many cases, the intrinsic activity of reported HEA can be increased by reducing the mixing entropy.

Lastly, with this hypothesis, we can revisit the reactions for which HEA are typically studied. These are the energy conversion reactions: HER, hydrogen oxidation reaction (HOR), and ORR. For the HER and HOR, Pt is the most active catalyst performing proton reduction at a negligible overpotential, severely complicating the assessment of its true intrinsic activity.^46–50^ This experimental evidence suggests that the activity of pure Pt sites is at a theoretical maximum. Therefore, attempts to enhance the activity of Pt sites through alloying would be constrained by the dilution effect. Similarly, if a Pt-free alloy produces complex sites with perfect activity, its lower surface contribution will result in a lower intrinsic activity than that of pure Pt. Consequently, this analysis suggests that it is impossible to outperform pure Pt for the HER and HOR.

In case of the ORR, the catalytic performance on heterogeneous metal surfaces is typically limited by the scaling relations between the adsorption energies of the catalytic intermediates.^51^ As a result, the theoretical optimum lies far from the thermodynamic equilibrium potential. Pt and Pd exhibit intermediate adsorption energies close to the theoretical optimum, making them the most active metal catalysts for the ORR. Thereby, Pt is reliably demonstrating the highest activity in the experimental setting.^42^ Consequently, in a composition space containing Pt or Pd, we anticipate that the most active ORR catalyst would be a Pt- or Pd-based low-entropy alloy. On the contrary, in spaces without Pt and Pd, the probability of a multielement alloy being the optimal catalyst increases. However, because statistical dilution still affects disordered surfaces, these alloys may outperform pure Pt but are unlikely to outperform optimized Pt-based binary alloys.

## Future of HEA Catalysis

The presented hypothesis examines the components of the intrinsic activity in disordered alloy structures, where the surface composition matches the bulk. It suggests that HEA catalysts face inherent limitations, making it challenging for them to exceed the activity of their low-entropy counterparts. Nevertheless, experimental evidence presents a more complex picture, with numerous reports describing HEAs as highly active catalysts. To address this discrepancy, we outline several properties of HEAs that remain understudied. Furthermore, we recommend that future research focus on these aspects and investigate them in greater detail.

First, we draw a distinction between the intrinsic activity and the effective, i.e., the observed, activity. The intrinsic activity refers to the fundamental catalytic performance of a catalyst in a kinetic regime and has been discussed so far in this perspective. This would be the theoretical maximum possible activity that the catalyst can possess. Analysis of this intrinsic activity is a necessary process to screen for promising candidates but is not sufficient to identify the optimal candidate. Namely, the optimal candidate needs to excel in the effective activity, which bears contributions originating from the environment in which the catalysis takes place. For instance, the effective activity may depend on the stability of the system, which is detrimental under ORR^52–54^ and OER^39,55^ conditions. Likewise, in systems where catalytic sites get poisoned by side-products, such as in the ethanol oxidation^56–58^ and formic acid oxidation,^59^ HEA sites may increase the resilience, ultimately leading to an enhanced activity. In this context, we recommend investigating HEAs for reactions in which the intrinsic activity is constrained by extrinsic contributions.

Second, this work employs the definition that the bulk and surface compositions of a HEA are identical. However, this assumption may not hold true under the experimental conditions. During operation, the surface of HEAs may leach out and segregate, producing core–shell structures with an active, low-entropic, high surface area, monometallic surface, and a HEA core.^60,61^ Such a restructuring would violate the employed definition and render the conclusions drawn from the statistical model inapplicable. Importantly, in this scenario, the catalyst derives its activity from a low-entropy surface rather than from the widely assumed cocktail effect of a multimetallic surface. Similarly, the entire HEA may dealloy into a highly porous framework. This porous structure can improve the mass transport properties, thereby enhancing the effective activity. In addition, the resulting porous architecture may exhibit a perturbed electronic structure, further increasing the intrinsic activity. This would be similar to dealloyed PtCuCo or PtCu exhibiting an enhanced activity for the ORR compared to Pt.^62^ In this scenario, HEA is completely replaced by a low-entropy structure whose activity originates from a perturbed electronic structure and a high surface area. Therefore, to advance the understanding of the activity of HEA catalysts, we recommend developing rigorous and robust methods for determining the electrochemically active surface area of disordered catalysts, paired with in situ and postelectrochemical characterization of the catalytic surface.

Continuing this discussion, the stability of HEA catalysts itself is an open question, and it is unclear which of the previously mentioned processes occur. For example, a HEA may exhibit high stability and preserve its disordered surface, potentially offsetting the cost in intrinsic activity. Alternatively, a HEA core–shell or dealloyed structure could prove more stable than a conventional low-entropy catalyst, making HEAs valuable precursors for catalyst engineering. All these possibilities represent major topics for future research.^61,63^

Furthermore, besides catalytic activity and stability, developing industrial catalysts involves a techno-economical aspect. It may be that a HEA catalyst is cheaper than the currently employed catalysts while delivering sufficient activity to serve as a viable alternative. For example, in the case of the ORR, the presented hypothesis does not exclude the possibility that a HEA catalyst could match the performance of pure Pt. Therefore, HEAs could be explored as a platform for reducing platinum-group-metal content in catalysts without compromising activity.

Concluding this work, using the theory and statistical modeling, we analyzed the surface complexity-activity relationship. We observe that increasing the activity of a metal by alloying is favorable only if the ligand interactions can compensate for the statistical dilution. This statistical dilution inevitably increases with system complexity, while the magnitude of the ligand interactions is limited by the chemistry of the material. As a result, we expect the intrinsic activity of HEA surfaces to be lower than those of their low-entropic components. Based on these considerations, we advise a more holistic approach to the field of HEA catalysis and to refocus its efforts from pursuing the global most active HEA catalyst to studying properties that are unique to HEA systems. These properties may contain but are not limited to stability and morphology control of the catalytic system.

## Supplementary Material



## Notes

Computational details of the statistical model. Elaboration of assumption A5 (PDF)
