# Supplementary material for: Unraveling the Mixing Entropy-Activity Relationship in High Entropy Alloy Catalysts: The More, The Better?
Source: J Am Chem Soc. 2026 Jan 26;148(5):4815–25. doi: 10.1021/jacs.5c15697 (PMC12903860; doi:10.1021/jacs.5c15697)
Supplement: Supplementary file 1 [file ja5c15697_si_001.pdf]

Supporting Information to: Unraveling the Mixing Entropy-Activity Relationship in High Entropy Alloy Catalysts: The More, The Better?

Vladislav A. Mints[a,c], Jack K. Pedersen[b], John C. Olsen[b], Mads K. Plenge[b], Matthias Arenz[c], Jan Rossmeisl[b]

[a] Department of Chemical Engineering, Imperial College London, Imperial College Rd, South Kensington, London SW7 2AZ, United Kingdom

[b] Center for High Entropy Alloy Catalysis (CHEAC), Department of Chemistry, University of Copenhagen, Universitetsparken 5, 2100 København Ø, Denmark.

[c] Department for Chemistry, Biochemistry and Pharmaceutical Sciences, University of Bern, Freiestrasse 3, 3012, Bern, Switzerland.

Corresponding authors: v.mints@imperial.ac.uk; matthias.arenz@unibe.ch; jan.rossmeisl@ku.dk

Computational Details of the Statistical Model

To avoid arithmetic errors in the statistical model, we used a python script to simulate all possible (5^6=15625) arrangements. Subsequently, using the rules described in the assumptions the active site distributions were calculated by counting the appropriate number of arrangements in the simulation. The source code to reproduce the calculations is available on https://github.com/vamints/HEA_statistical_model

In the code, the function *get_break_even_activities()* calculates active site distributions and minimum activities that active sites need to posses for the alloy to be competitive. The function takes 4 input parameters: *compositions*, *permutations, c_effective_distribution*, *c_dead_surface*. *compositions* is an array with the shape (5,5) that contains the 1-element to 5-element compositions for which the break even activities are evaluated. *permutations* is a pandas dataframe that contains the 15625 simulated 6-atom environments. *c_effective_distribution* is a tuning parameter used to combat the combinatorial effect in the statistical model as stated by Assumption A5. In this work *c_effective_distribution*=0 to avoid introducing artifacts from combinatorics. By stetting the value to 1, all sites irrespective of the combination are considered equally active. *c_dead_surface* influences assumption A4 by tuning the relative activity of active sites with the second element (m_2) in the center. Following assumption A4 the value is set to 0.68. By setting *c_dead_surface* to 0 active sites with m_2 can be completely switched off. The code contains a grid optimizer to find the compositions that full fill assumption A5. Lastly, the code contains a Bayesian Optimizer to search for the most active composition based on this model and given a set of active-site activities.

The function *get_break_even_activities()* returns 3 tables (Table S1-3). These tables are showing the data from Figures 5,6 in the main text. Table S1, summarizes the considered active site distributions. Table S2, contains the surface loss due to the alloying process. For instance, when going from a pure metal to a 5-element alloy, 100% of the surface is lost. Likewise, when going from a 2-element alloy to a 5-element alloy 84% of the surface devoted to 2-element sites is invested. Based on the data in Table S1 and S2 the minimum activities a complex site needs to exhibit can be calculated. The calculation is: the amount of surface lost divided by the amount of surface received for the most complex site.

Table S1. The active site distribution calculated based on the statistical model outlined in the main text. This is achieved with parameters *c_effective_distribution* = 0 and *c_dead_surface = 0.68*. The headers show the optimized composition for which the active site distribution is calculated.

	**[1, 0, 0, 0, 0]**	**[0.6, 0.4, 0, 0, 0]**	**[0.41, 0.33, 0.26, 0, 0]**	**[0.30, 0.26, 0.22, 0.22, 0]**	**[0.22, 0.22, 0.18, 0.19, 0.19]**
**1-element**	1.000	0.029	0.002	0.000	0.000
**2-element**		0.843	0.102	0.014	0.002
**3-element**			0.486	0.085	0.017
**4-element**				0.223	0.050
**5-element**					0.073

Table S2. The fraction of lost surface due to the alloying process based on the values in Table S1.

	**Binary alloy**	**Ternary alloy**	**Quaternary alloy**	**Quinary alloy**
**1-element**	0.971	0.998	1.000	1.000
**2-element**		0.741	0.828	0.841
**3-element**			0.401	0.469
**4-element**				0.172

Table S3. Minimum activities that complex sites need to possess for an alloy to outperform their less complex counterparts based on data in Table S1 and S2.

	Binary alloy	Ternary alloy	Quaternary alloy	Quinary alloy
**1-element**	1.2	2.1	4.5	13.6
**2-element**		1.5	3.7	11.5
**3-element**			1.8	6.4
**4-element**				2.3

Binary Alloy activities for ORR that 5-element alloys cannot outperform

The calculation of the activity of a binary alloy that a 5-element alloy cannot outperform requires the consideration that all sites may have an activity. Thus, if all 5-element sites are ideal, the baseline of the 5-element alloy would be an ORR activity of 3.5 Pt eq. Yet, if the binary, ternary and quaternary sites have an activity larger than Pt, the total activity will be higher. The maximum activity at which the 5-element sites will become redundant can be calculated in an iterative way backwards using the different minimum activities. This means that 5-element sites cannot outperform 4-element sites with an activity that is larger than 48/2.35 = 20.4. Continuing down the line, they cannot outperform 3-element sites with an activity of 20.4/1.8 = 11.4 and 2-element sites with an activity of 7.5. To transform this into an activity of an alloy, we can assume that only these sites are active. Thus, a binary alloy will have a minimum activity of approximately 7.5*0.84 = 6.4 and a ternary alloy will have a minimum activity of 11.4*0.49 = 5.6.

Artifact Mitigation through Assumption A5

The magnitude of assumption A5 can be tuned using the parameter *c_effective_distribution* in the code. This assumption is necessary to combat the combinatorial effect of going up in dimensions, which is easiest explained using examples. Let's consider the case where *c_effective_distribution* = 1 and for simplicity we assume that *c_dead_surface* = 0, thus only sites with m_1 in the center are active. In this system a 2-element alloy is described by 2 types of active sites: 1-element sites (m_1) and 2-element sites (m_1m_2). When creating a 3-element alloy you now get 3 types of active sites 1-element sites (m_1), 2-element sites ($m_1m_2 + m_1m_3$), and 3-element sites ($m_1m_2m_3$). If we now assume that m_1m_2 and m_1m_3 are identical we can substitute m_2 with m_3 without having to pay any price. As a result, as long as $m_1m_2m_3$ is more active than m_1m_2 it is beneficial to create a 3-element alloy. The same logic applies further down the line to the 5-element system. As long as the 5-element sites are more active it will be beneficial to do some form of substitution as no price is being paid to do that. Furthermore, assuming *c_effective_distribution* = 1 also introduces a combinatorial artifact when going up in dimensions. It is most pronounced when going from 4-elements to 5-elements. While the probability of finding a specific 4-element combination goes down a 5-element space has 4 different 4-element sites with m_1 in the center. Consequently, the effective fraction of 4-element sites barely decreases due to the alloying process essentially making it almost always beneficial to go from 4-elements up to 5-elements.

Summarizing, changing *c_effective_distribution* only affects the effective surface area of the less complex sites in an alloy. It is most affecting very complex systems with 5 and more elements. This is illustrated in Figure S1 and S2. These Figures show how the active site distribution and minimum activity change by changing *c_effective_distribution*. Most minimum activities are unaffected by this except the minimum activity that 5-element sites need to possess when competing with 4-element sites.

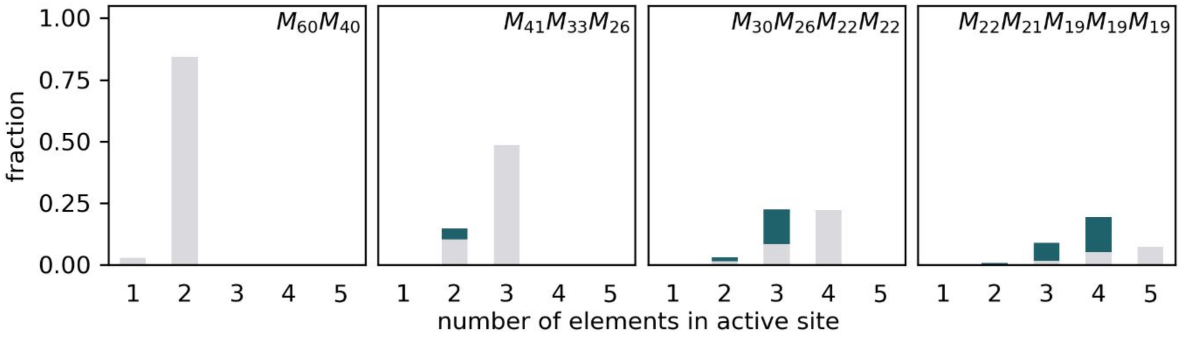

Fig S1. The different active site distributions using (light grey) *c_effective_distribution* = 0 and (dark grey) *c_effective_distribution* = 1 and *c_dead_surface* = 0.68.

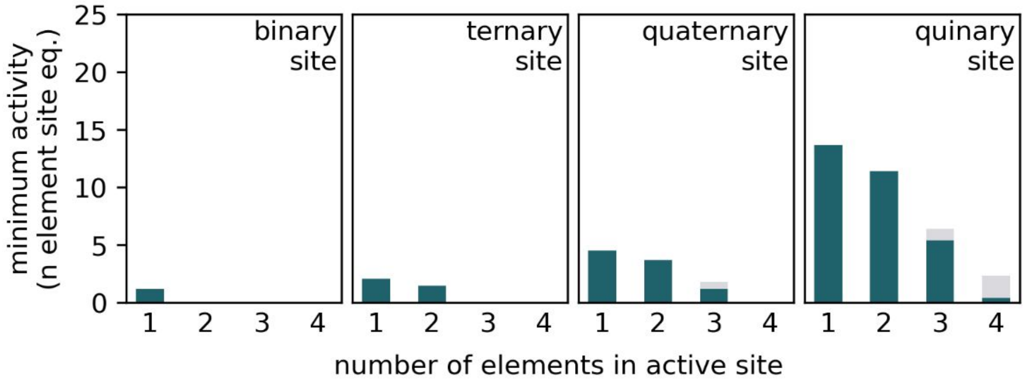

Fig S2. The difference in minimum activities using (light grey) *c_effective_distribution* = 0 and (dark grey) *c_effective_distribution* = 1 and *c_dead_surface* = 0.68.
